# Possible epigenetic regulatory effect of dysregulated circular RNAs in Alzheimer’s disease model

**DOI:** 10.1038/s41598-019-48471-z

**Published:** 2019-08-16

**Authors:** Woo-Jin Lee, Jangsup Moon, Daejong Jeon, Yong-Won Shin, Jung-Suk Yoo, Dong-Kyu Park, Soon-Tae Lee, Keun-Hwa Jung, Kyung-Il Park, Ki-Young Jung, Manho Kim, Sang Kun Lee, Kon Chu

**Affiliations:** 1 Department of Neurology, Comprehensive Epilepsy Center, Laboratory for Neurotherapeutics, Biomedical Research Institute, Seoul National University Hospital, Seoul, South Korea; 2 Program in Neuroscience, Neuroscience Research Institute of SNUMRC, College of Medicine, Seoul National University, Seoul, South Korea; 3 Advanced Neural Technologies, Seoul, South Korea; 4 Department of Neurology, Seoul National University Healthcare System Gangnam Center, Seoul, South Korea

**Keywords:** Cognitive ageing, Alzheimer's disease, Epigenetics and behaviour

## Abstract

As circular RNAs (circRNAs) regulates the effect of micro RNAs (miRNAs), circRNA–miRNA-mRNA network might be implicated in various disease pathogenesis. Therefore, we evaluated the dysregulated circRNAs in the Tg2576 mouse Alzheimer’s disease (AD) model, their possible regulatory effects on downstream target mRNAs, and their pathomechanistic role during the disease progression. The microarray-based circRNA expression analysis at seven- and twelve-months of ages (7 M and 12 M) returned 101 dysregulated circRNAs at 7 M (55 up-regulated and 46 down-regulated) and twelve dysregulated circRNAs at 12 M (five up-regulated and seven down-regulated). For each dysregulated circRNA, potential target miRNAs and their downstream target mRNAs were searched. Dysregulation of circRNAs was associated with increased frequency of relevant dysregulation of their downstream target mRNAs. Those differentially expressed circRNA–miRNA-mRNA regulatory network included 2,275 networks (876 for up-regulated circRNAs and 1,399 for down-regulated circRNAs) at 7 M and 38 networks (25 for up-regulated circRNAs and 13 for down-regulated circRNAs) at 12 M. Gene ontology (GO) and pathway analyses demonstrated that the dysregulated mRNAs in those networks represent the AD pathomechanism at each disease stage. We concluded that the dysregulated circRNAs might involve in the AD pathogenesis by modulating disease relevant mRNAs via circRNA–miRNA-mRNA regulatory networks.

## Introduction

Alzheimer’s disease (AD) is a progressive age-related disease in the brain characterized by accumulation of amyloid plaques, formation of neurofibrillary tangles, synaptic dysfunction, and neuronal degeneration^1–3^. At each stages in the AD pathogenesis, genes are tightly regulated by various epigenetic regulatory mechanisms such as DNA methylation, histone modification, and regulation by noncoding RNAs (ncRNAs)^2,4–7^. Among them, the role of micro RNAs (miRNAs), small (20–24 bp) non-coding RNAs (ncRNAs) that bind to the target mRNAs to direct their repression, has been most extensively investigated^2,8–15^.

As miRNAs are implicated in the fine tuning of gene expression, the regulation of miRNA expression in the brain is highly dynamic and complex. MiRNAs are known to alter their expression levels within 90 minutes and their half-life in brain is generally less than 3.5 hours^16,17^. MiRNAs regulate the hundreds of target mRNAs and mRNAs are also targeted by hundreds of miRNAs^18,19^. Therefore, a solitary dysregulation of miRNAs in a specific time-point might not be sufficient to take charge of the sustained dysregulation of pathophysiologically relevant genes into a long-term progression of AD.

Growing evidence suggest that gene expression is more precisely modulated at a higher level of complexity by a distinct type of ncRNA, called circular RNA (circRNA). CircRNA is a covalently closed and circular-shaped subgroup of ncRNAs generated by back-splicing process^20,21^. CircRNAs are increasingly recognized as major epigenetic regulators in the various disease pathogenesis via several mechanisms^20–24^. First, the production of circRNA might complete with that of the corresponding linear mRNAs. Second, circRNAs regulates the expression of gene transcription machineries. Most importantly, circRNAs contains multiple binding sites for miRNAs that enable to sequestrate or buffer the effect of the target miRNA. In this regard, circRNA might modulate the expression of their target genes by participating in circRNA-miRNA-mRNA regulatory network^25–27^.

Some properties of circRNA indicate that circRNAs might have a particular role in the pathogenesis of AD. First, the regulation of circRNA expression shows a time- and region-specific pattern and is independent from that of the corresponding linear-form mRNAs^28,29^. Second, circRNAs are highly abundant and stable in the brain due to their closed loop structures making them resistant to RNA exonucleases or RNase R-mediated degradation^21,29^. Therefore, circRNAs can consistently buffer the highly fluctuating effect of miRNA and their altered expression might sufficiently direct the overall gene expression profile into a certain disease process via circRNA-miRNA-mRNA networks^22^. In this context, studying the altered profile of circRNA expression in different disease stages and its implication on the downstream target mRNAs might help elucidate a novel epigenetic pathomechanism of AD.

In this study, we hypothesized that changes in circRNA expression during the AD progression might be implicated in the pathogenesis of AD by epigenetic regulation via circRNA-miRNA-mRNA network. Therefore, we investigated the association among the dysregulated circRNAs and the altered expression profiles of their downstream target miRNAs and mRNAs in different disease stages of AD by constructing circRNA-miRNA-mRNA network based on the microarray database and evaluated their potential role in the pathogenesis of AD by bioinformatics analyses.

## Results

### Overall expression profile of mRNA, miRNA, and circRNA

**Figure 1:**
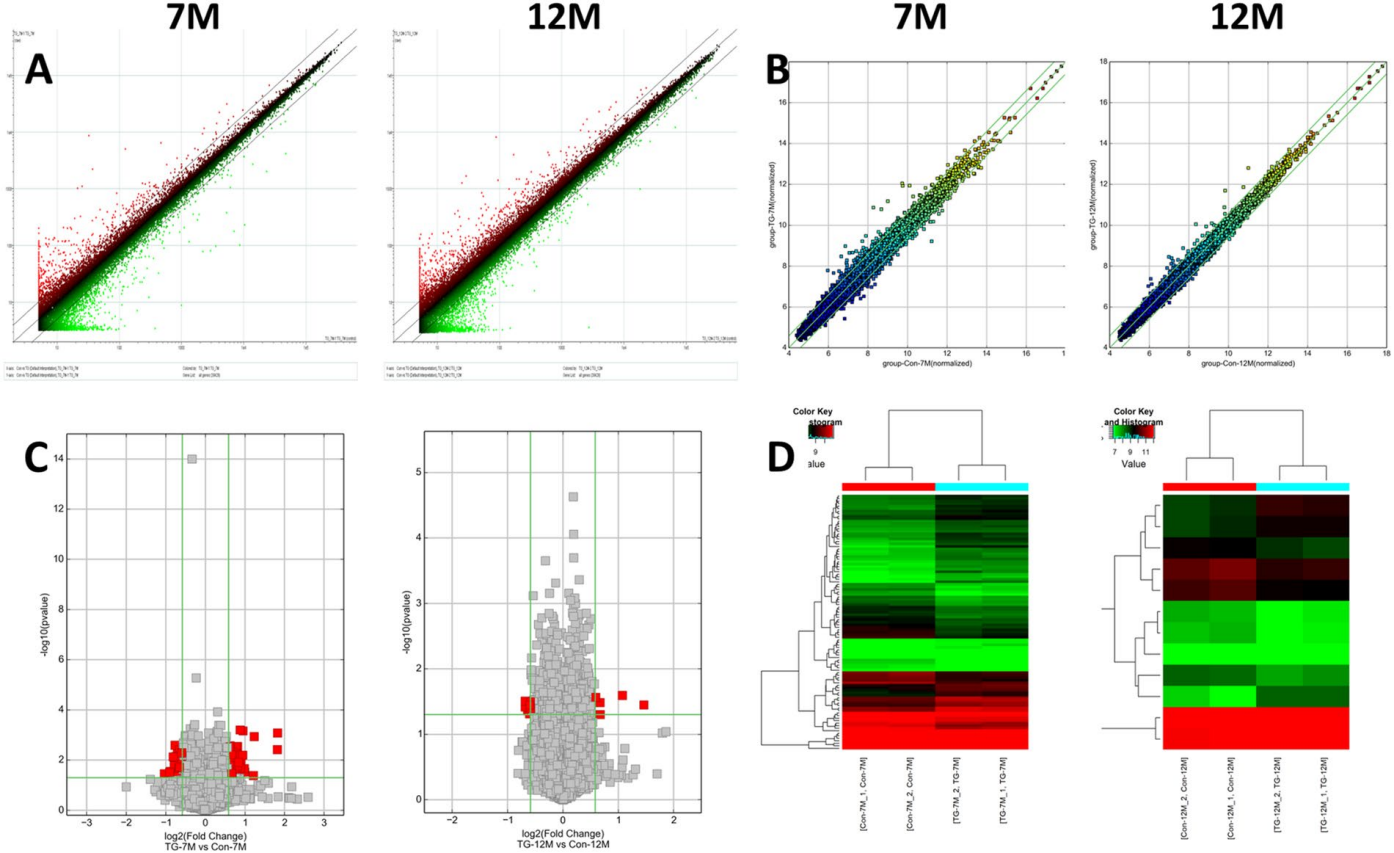
Microarray data analysis of the differentially expressed mRNAs and circRNAs in the brain of Alzheimer’s disease model. Scatter Plot (panel A) shows the distribution of the expression levels of mRNAs in the Alzheimer’s disease (AD) model and the control. Scatter Plot (panel B) and volcano plot (panel C) demonstrate the distribution of the expression levels of circRNAs in the AD model and the control. The green lines in scatter plot and volcano plot demark the fold change of 1.5. The X- and Y-axes in the scatter plot indicate the averaged normalized signal values of the group (log2 scaled). In the volcano plot, red squares represent the differentially expressed circRNAs (*P*-value < 0.05). Panel D shows hierarchical cluster analysis of differentially expressed circRNAs. The log2 signal intensity is reflected in the color scale, which runs from green (low intensity) to red (strong intensity).

**Figure 2:**
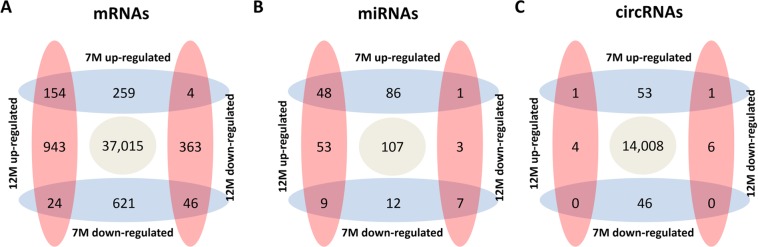
Diagram of differentially expressed mRNAs, miRNAs, and circRNAs. Panels A–C demonstrate the numbers of up- or down-regulated mRNAs, miRNAs, and circRNAs at each time point, respectively.

Among a total of 39,429 mRNAs analyzed, the number of differentially expressed mRNAs in the brain of 7 M Tg2576 mouse was 1,108 (up-regulated, 417; down-regulated, 691) and 12 M mouse was 1,534 (up-regulated, 1,121; down-regulated, 413). Scatter plots for the mRNA expression pattern were demonstrated in Fig. 1A. In most cases, dysregulation of mRNAs was time specific, not persisting or being reversed between 7 M and 12 M (2186/2414, 90.6%, Fig. 2A). Top five most dysregulated mRNAs in 7 M and 12 M Tg2576 mice were listed in Table 1. The full data of mRNAs expression is available in Supplemental Data S1.

**Table 1:** Five most highly dysregulated circRNAs in Alzheimer’s disease model.

circRNA name	Type^*^	Best related linear transcript (Gene symbol)	Fold change	*P* value
**7 M Up-regulated**				
mmu_circRNA_28972	exonic	NM_025730 (Lrrk2)	3.540	0.001
mmu_circRNA_28971	exonic	NM_025730 (Lrrk2)	3.528	0.004
mmu_circRNA_29980	overlapping	NM_007471 (App)	2.351	0.001
mmu_circRNA_35502	exonic	NM_019918 (Vmn2r1)	2.320	0.041
mmu_circRNA_39081	exonic	NM_010097 (Sparcl1)	1.992	0.030
**7 M Down-regulated**				
mmu_circRNA_22066	exonic	NM_172788 (Sh3rf3)	2.056	0.035
mmu_circRNA_001769	exonic	NR_046233 (Rn45s)	1.872	0.030
mmu_circRNA_30284	overlapping	NM_001104569 (Vmn2r107)	1.844	0.028
mmu_circRNA_011516	exonic	ENSMUST00000023060 (Npcd)	1.823	0.037
mmu_circRNA_19403	exonic	ENSMUST00000050472 (Uspl1)	1.758	0.007
**12 M Up-regulated**				
mmu_circRNA_29980	intronic	NM_007471 (App)	2.753	0.035
mmu_circRNA_45982	exonic	NM_010797 (Mid1)	2.102	0.025
mmu_circRNA_23412	exonic	NM_028451 (Larp1)	1.594	0.049
mmu_circRNA_19523	intronic	NM_172536 (Zfp609)	1.590	0.032
mmu_circRNA_012412	overlapping	NR_046233 (Rn45s)	1.508	0.027
**12 M Down-regulated**				
mmu_circRNA_34884	exonic	NM_001291137 (Ralgapb)	1.602	0.038
mmu_circRNA_008816	exonic	NM_027436 (Mipep)	1.601	0.031
mmu_circRNA_37345	exonic	NM_011327 (Scp2)	1.556	0.043
mmu_circRNA_45069	exonic	NM_153413 (Dock3)	1.516	0.048
mmu_circRNA_35843	exonic	NM_013841 (Vps45)	1.512	0.035

^*^Exonic represents circRNA arising from the exons of the linear transcript, intronic the circRNA arising from an intron of the linear transcript, and overlapping the circRNAs transcribed from same gene locus as the linear transcript, but not classified into exonic or intronic.

The total number of analyzed miRNAs was 326. Among them, the number of differentially expressed miRNAs in the brain of 7 M Tg2576 mouse was 163 (up-regulated, 135; down-regulated, 28) and 12 M mouse was 121 (up-regulated, 110; down-regulated, 11). The majority of the miRNA dysregulation was time specific (154/219, 70.3%, Fig. 2B). The full data of miRNA expression is demonstrated in Supplemental Data S2.

The total number of analyzed circRNAs was 14,119. Among them, the number of differentially expressed circRNAs in the brain of 7 M Tg2576 mouse was 101 (up-regulated, 55; down-regulated, 46) and 12 M mouse was 12 (up-regulated, 5; down-regulated, 7). Scatter plots, volcano plots, and hierarchical clustering for the circRNA expression pattern were demonstrated in Fig. 1B–D. Most of the dysregulation of circRNAs was also time specific (109/111, 98.2%, Fig. 2C). Top five most highly dysregulated circRNAs in 7 M and 12 M Tg2576 mice were listed in Table 2. The full data of circRNA expression is in the Supplemental Data S3.

**Table 2:** Five most highly dysregulated mRNAs in Alzheimer’s disease model.

Name	Transcript	Fold change	*P* value
**7 M Up-regulated**			
Cst7	NM_009977	158.445	0.012
Itgax	NM_021334	35.916	0.018
Ccl4	NM_013652	30.387	0.019
Apoc4	NM_007385	15.836	0.002
Gm9992	NM_001142539	10.776	0.001
**7 M Down-regulated**			
Fer1l4	NM_001136556	17.811	0.024
Ms4a15	NM_001034898	16.922	0.012
Olfr155	NM_019473	15.691	0.032
Il13	NM_008355	15.619	0.026
Dep1	AF032130	14.775	0.015
**12 M Up-regulated**			
Cst7	NM_009977	90.983	<0.001
Itgax	NM_021334	56.652	<0.001
Ctse	NM_007799	31.268	0.085
Clec7a	NM_020008	25.360	<0.001
Ccl3	NM_011337	18.862	0.004
**12 M Down-regulated**			
Athl1	AK220240	100.002	<0.001
LOC102640520	XR_880780	25.837	0.004
Olfr1261	NM_146474	20.713	0.018
Gm14296	NR_130970	15.735	0.369
Olfr698	NM_146602	12.560	0.025

### PCR analysis of circRNA and mRNA expression

**Figure 3:**
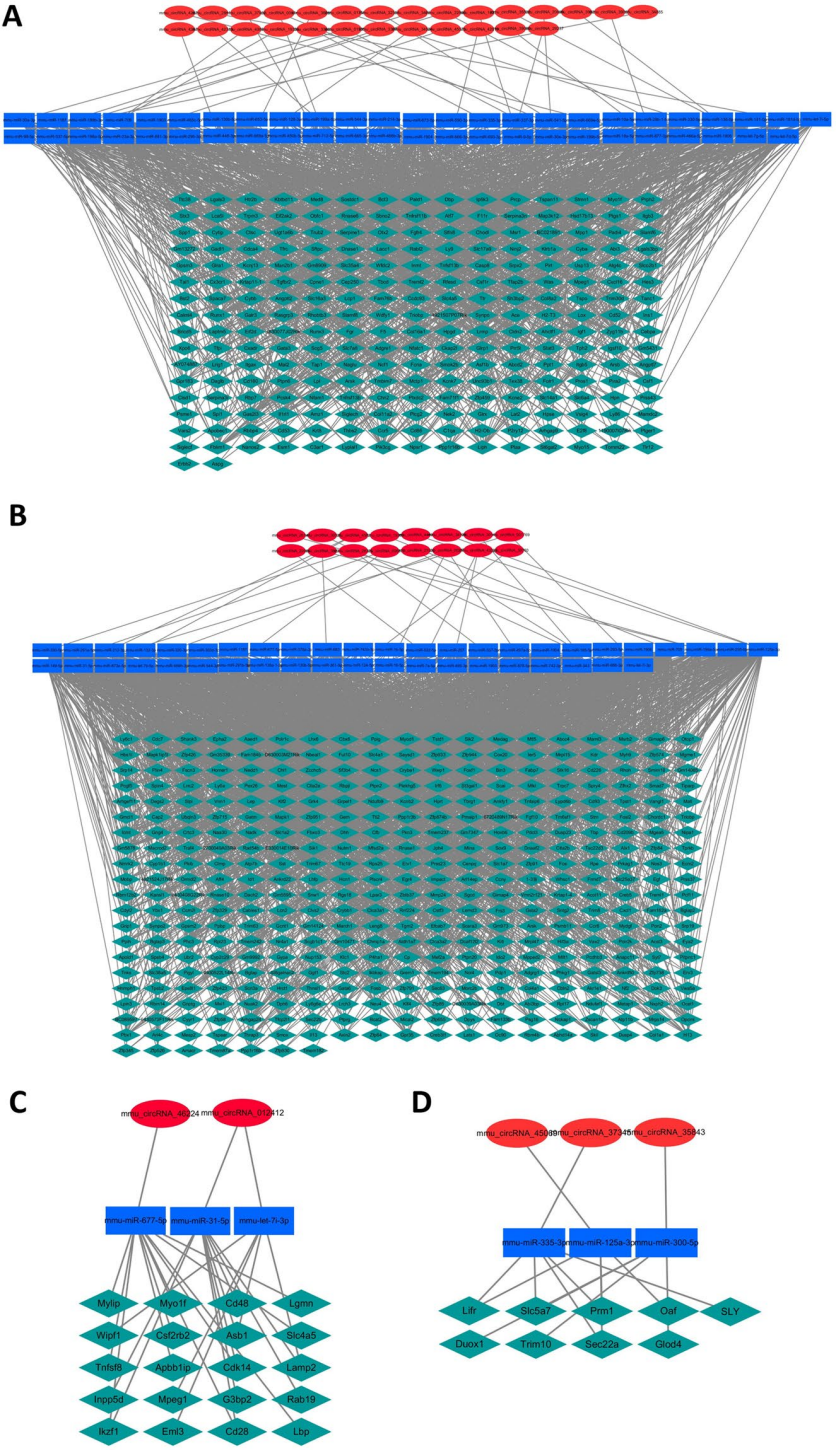
Differentially expressed circRNA–miRNA–mRNA regulatory network in the brain of Alzheimer’s disease model. For the 55 up-regulated circRNAs (red ellipses) and 23 downstream target miRNAs (blue rectangles) in 7 M brain, 242 downstream mRNAs (green diamonds) were relevantly up-regulated, consisting a total of 876 circRNA–miRNA–mRNA regulatory network (panel A). For the 46 down-regulated circRNAs and 21 downstream target miRNAs in 7 M brain, 388 downstream mRNAs were relevantly down-regulated, consisting a total of 1,399 circRNA–miRNA–mRNA regulatory network (panel B). For the 5 up-regulated circRNAs and 3 downstream target miRNAs in 12 M brain, 20 downstream mRNAs were relevantly up-regulated, consisting a total of 25 circRNA–miRNA–mRNA regulatory network (panel C). For the 7 down-regulated circRNAs and 3 downstream target miRNAs in 12 M brain, 9 downstream mRNAs were relevantly down-regulated, consisting a total of 13 circRNA–miRNA–mRNA regulatory network (panel D).

Three RNAs were randomly selected from each of the top ten (five up-regulated and five down-regulated) most highly dysregulated circRNAs and mRNAs in 7 M and 12 M Tg2576 mice. Quantitative polymerase chain reaction (PCR) analysis of those 12 RNAs (six circRNAs and six mRNAs) was performed using the primers described in the Supplemental Data S4. As the result, 5/6 (83.3%) circRNAs and 5/6 (83.3%) mRNAs were confirmed to be differentially expressed relevantly to the microarray data (Fig. 3, see Supplemental Data S5 for the PCR raw data).

### Analysis of circRNA–miRNA–mRNA regulatory interaction

First, we evaluated the regulatory effect of miRNAs on their target mRNAs, separately for the up- and down-regulated miRNAs in each time point (7 M and 12 M). 10,243 mRNAs were identified as the potential targets for the 135 up-regulated miRNAs in the 7 M brain, 3,100 mRNAs for the 28 down-regulated miRNAs in the 7 M brain, 9,475 mRNAs for the 110 up-regulated miRNAs in the 12 M brain, and 1,587 mRNAs for the 11 down-regulated miRNAs in 12 M the brain. However, no significantly higher frequency of dysregulation was observed in the downstream target mRNAs of the differentially expressed miRNAs (Table 3A). Rather, the frequencies of down-regulation in the target mRNAs of up-regulated miRNAs in the 12 M brain and up-regulation in the target mRNAs of down-regulated miRNAs in the 7 M brain were significantly low.

**Table 3:** Chi-square analyses for the frequency of relevant dysregulation in the downstream target RNAs.

Targeted by dysregulated upstream miRNAs	Dysregulation of downstream mRNAs	Odds Ratio^†^	95% Confidence interval	*P* value
**A**				
7 M up-regulated miRNAs	Down-regulation	1.123	0.950–1.327	0.175
7 M down-regulated miRNAs	Up-regulation	0.495	0.304–0.806	0.004^*^
12 M up-regulated miRNAs	Down-regulation	0.722	0.563–0.926	0.010^*^
12 M down-regulated miRNAs	Up-regulation	0.763	0.543–1.073	0.119
**Targeted by dysregulated upstream circRNAs**	**Dysregulation of downstream miRNAs**	**Odds Ratio**	**95% Confidence interval**	***P*** **value**
**B**				
7 M up-regulated circRNAs	Down-regulation	0.484	0.063–3.739	0.478
7 M down-regulated circRNAs	Up-regulation	1.065	0.436–2.604	0.889
12 M up-regulated circRNAs	Down-regulation	—	—	1.000
12 M down-regulated circRNAs	Up-regulation	4.037	0.362–45.025	0.260

^†^Odds ratios for the frequency of significant dysregulation in the downstream RNAs, according to whether being targeted by the upstream RNAs, *P < 0.05, and **P < 0.01.

Second, we evaluated the regulatory effect of differentially expressed circRNAs on the expression of their target miRNAs. From the MRE sequence analyses, 205 miRNAs were identified as the potential targets for the 55 up-regulated circRNAs in the 7 M brain, 184 miRNAs for the 46 down-regulated circRNAs in the 7 M brain, 27 miRNAs for the 5 up-regulated circRNAs in the 12 M brain, and 32 miRNAs for the 7 down-regulated circRNAs in the 12 M brain. From the microarray data of 326 miRNAs, the expression of 23/205, 21/184, 3/27, and 3/32 of those potential target miRNAs, respectively, were identified. However, no significantly altered frequency of dysregulation was observed in the downstream target miRNAs of the differentially expressed circRNAs, supporting that the regulatory effect of circRNAs on their target miRNAs does not result in the alteration of the target miRNA expression level (Table 3B).

Third, we evaluated the regulatory effect of differentially expressed circRNA–miRNA–mRNA regulatory networks on their target mRNAs. 5,406 mRNAs were identified as the potential targets for the 23 target miRNAs of the 55 up-regulated circRNAs in the 7 M brain, 5,266 mRNAs for the 21 target miRNAs of the 46 down-regulated circRNAs in the 7 M brain, 787 mRNAs for the 3 target miRNAs of the 5 up-regulated circRNAs in the 12 M brain, and 65 mRNAs for the 3 target miRNAs of the 7 down-regulated circRNAs in the 12 M brain. Notably, the frequency of dysregulation in the relevant direction of the downstream target mRNAs was significantly high in every group of different time and direction of upstream circRNA expression (Table 4A). After adjusting the effect of dysregulated upstream miRNAs, the altered expression of upstream circRNAs was significantly associated with the higher frequency of relevant dysregulation in downstream target mRNAs (*P* < 0.001 for up-regulated circRNAs in 7 M, down-regulated circRNAs in 7 M, and down-regulated circRNAs at 12 M and *P* = 0.039 for up-regulated circRNAs at 12 M, Table 4B).

**Table 4:** Analyses for the effect of dysregulated circRNAs on the expression of their downstream target mRNAs.

Targeted by dysregulated upstream circRNAs	Dysregulation of downstream mRNAs	Odds Ratio	95% Confidence interval	*P* value
**A**				
7 M up-regulated circRNAs	Up-regulation	8.535	7.017–10.381	<0.001^**^
7 M down-regulated circRNAs	Down-regulation	6.977	5.993–8.123	<0.001^**^
12 M up-regulated circRNAs	Up-regulation	1.413	1.049–1.903	0.039^*^
12 M down-regulated circRNAs	Down-regulation	17.578	8.897–34.727	<0.001^**^
**Dysregulation of mRNAs**		**Odds Ratio**	**95% Confidence interval**	***P*** **value**
**B**				
7 M up-regulation	Targeted by 7 M up-regulated circRNAs	12.425	10.178–15.169	<0.001^**^
7 M down-regulation	Targeted by 7 M down-regulated circRNAs	37.279	30.275–45.903	<0.001^**^
12 M up-regulation	Targeted by 12 M up-regulated circRNAs	1.413	1.049–1.903	0.039^*^
12 M down-regulation	Targeted by 12 M down-regulated circRNAs	22.081	10.996–44.344	<0.001^**^

Univariate (chi-square) analyses were summarized in Table A. In Table B, logistic analyses demonstrate the effect of dysregulated circRNAs on the relevant dysregulation of their downstream target mRNAs, after adjusting the effects of dysregulated miRNAs. *P < 0.05 and **P < 0.01.

### Differentially expressed circRNA–miRNA–mRNA regulatory network

**Figure 4:**
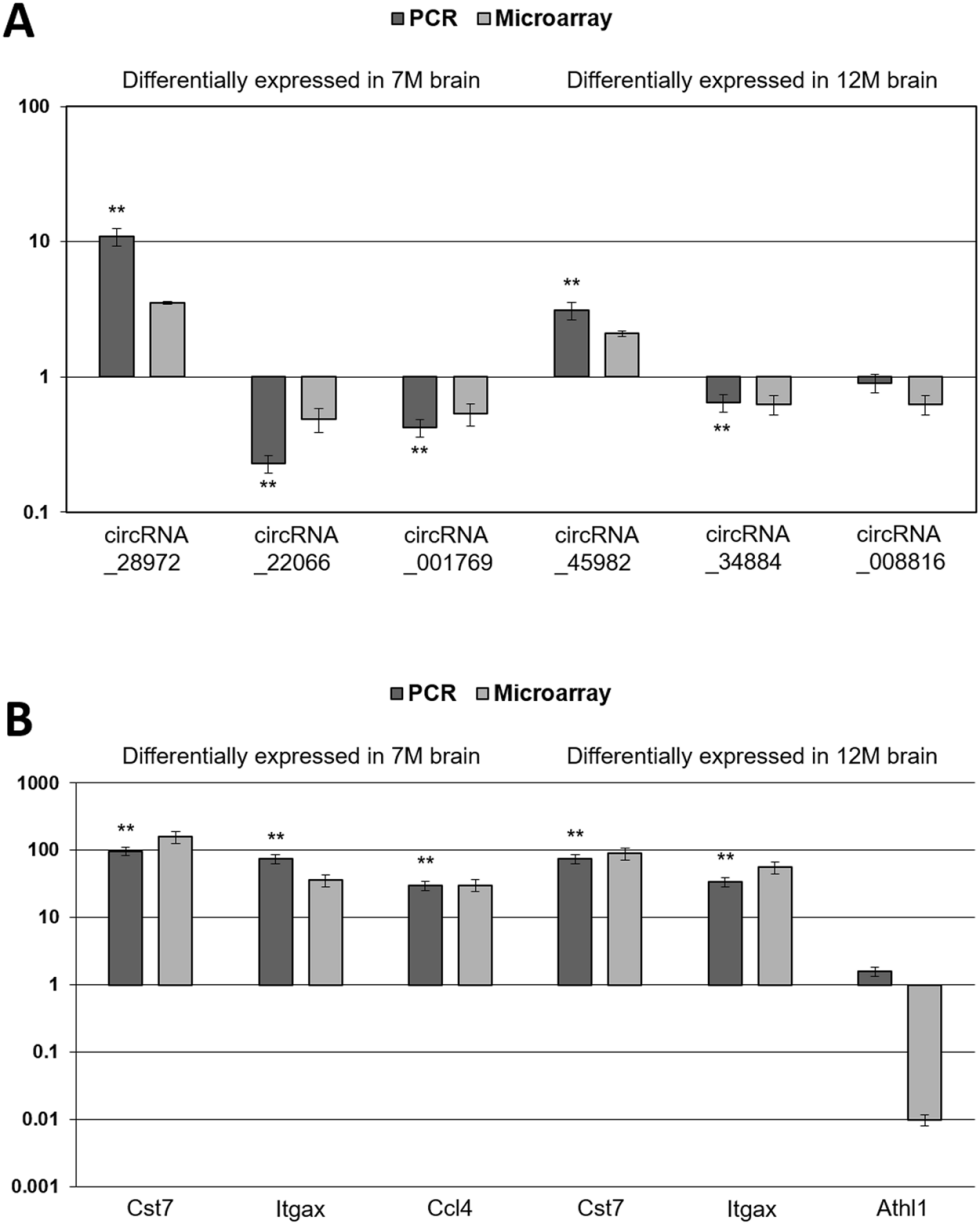
PCR validation of most highly dysregulated circRNAs and mRNAs. Log-transformed expression ratios of the circRNAs (panel A) and mRNAs (panel B) from the PCR analysis and microarray are demonstrated. Gapdh was used as a reference gene to calculate the expression ratios (Alzheimer’s disease model/control). Horizontal bars indicate the standard errors. *P < 0.05, **P < 0.01 for the statistical significance of altered expression.

Construction of the circRNA–miRNA–mRNA regulatory networks was performed separately for the up- and down-regulated circRNAs in each time point (7 M and 12 M). For the 55 up-regulated circRNAs and 23 downstream target miRNAs in 7 M brain, 242 downstream mRNAs were relevantly up-regulated, consisting a total of 876 circRNA–miRNA–mRNA regulatory network (Fig. 4A). For the 46 down-regulated circRNAs and 21 downstream target miRNAs in 7 M brain, 388 downstream mRNAs were relevantly down-regulated, consisting a total of 1,399 circRNA–miRNA–mRNA regulatory network (Fig. 4B).

For the 5 up-regulated circRNAs and 3 downstream target miRNAs in 12 M brain, 20 downstream mRNAs were relevantly up-regulated, consisting a total of 25 circRNA–miRNA–mRNA regulatory network (Fig. 4C). For the 7 down-regulated circRNAs and 3 downstream target miRNAs in 12 M brain, 9 downstream mRNAs were relevantly down-regulated, consisting a total of 13 circRNA–miRNA–mRNA regulatory network (Fig. 4D). The full list of differentially expressed circRNA–miRNA–mRNA regulatory network is in the Supplemental Data S6).

Additionally, a substantial complexity was observed in the differentially expressed circRNA–miRNA–mRNA regulatory networks, in which each miRNA was targeted by up to three upstream circRNAs (median 1, interquartile range, IQR 1−1), targeted up to 123 downstream mRNAs (median 32, IQR 18−54.25), and each mRNA was targeted by up to 9 upstream miRNAs (median 2.5, IQR 1−7).

### Gene ontology and pathway analysis

Gene ontology and pathway analyses were performed separately for the up- and down-regulated circRNA–miRNA–mRNA regulatory networks in each time-point. In each analyses, the top five enriched GO terms and KEGG pathways were summarized in Tables 5 (up-regulated mRNAs) and 6 (down-regulated mRNAs, full list of the enriched GO terms and KEGG pathways in Supplemental Data S7).

**Table 5:** Five most enriched gene ontology (GO) processes and KEGG pathway of up-regulated mRNAs in Alzheimer’s disease model.

7 months			12 months		
GO biological process	Fold Enrichment	P value	GO biological process	Fold Enrichment	P value
Macrophage colony-stimulating factor signaling pathway	70.1	0.028	T cell activation via T cell receptor contact with antigen bound to MHC molecule on APC	69.3	0.028
Cellular response to L-glutamine	46.7	0.042	Negative regulation of myeloid dendritic cell activation	52.0	0.038
Positive regulation of serotonin secretion	46.7	0.042	Positive regulation of tumor necrosis factor secretion	52.0	0.038
Positive regulation of thyroid hormone generation	46.7	0.042	Negative regulation of natural killer cell activation	52.0	0.038
Response to aldosterone	46.7	0.042	Positive regulation of interleukin-1 production	41.6	0.047
**GO molecular function**			**GO molecular function**		
Fc-gamma receptor I complex binding	49.5	0.040	C-X3-C chemokine binding	41.8	0.047
Beta-glucuronidase activity	37.1	0.033	Transforming growth factor beta-activated receptor activity	34.8	0.006
Superoxide-generating NADPH oxidase activity	27.8	0.005	Lipoteichoic acid binding	34.8	0.006
Enhancer sequence-specific DNA binding	8.6	0.047	Fibronectin binding	10.8	0.031
RNA polymerase II transcription factor binding	6.7	0.006	—	—	—
**GO cellular component**			**GO cellular component**		
CSF1-CSF1R complex	71.2	0.028	integrin complex	16.6	0.002
Alphav-beta3 integrin-IGF-1-IGF1R complex	35.6	0.055	immunological synapse	15.3	0.020
NADPH oxidase complex	23.7	0.007	phagocytic vesicle membrane	8.9	0.010
Sperm midpiece	10.7	0.031	receptor complex	5.4	0.002
Integrin complex	8.5	0.047	external side of plasma membrane	3.6	0.001
**Pathway analysis**			**Pathway analysis**		
Glycosaminoglycan degradation	9.5	0.038	Graft-versus-host disease	37.4	0.028
Complement and coagulation cascades	7.0	<0.001	Allograft rejection	34.4	0.038
Osteoclast differentiation	6.9	<0.001	Type I diabetes mellitus	31	0.042
Staphylococcus aureus infection	6.7	0.006	Intestinal immune network for IgA production	29.6	0.047
Hematopoietic cell lineage	6.3	<0.001	—	—	—

**Table 6:** Five most enriched gene ontology (GO) processes and KEGG pathway of down-regulated mRNAs in Alzheimer’s disease model.

7 months			12 months		
GO biological process	Fold Enrichment	P value	GO biological process	Fold Enrichment	P value
Muscle cell fate commitment	49.3	0.040	Cytokine-mediated signaling pathway	0.026	42.1
Negative regulation of muscle hyperplasia	49.3	0.040	Mesenchymal to epithelial transition involved in metanephros morphogenesis	0.027	31.8
Schwann cell proliferation	32.8	0.030	Positive regulation of action potential	0.042	25.8
Regulation of store-operated calcium entry	14.8	0.017	—	—	—
Mitochondrial genome maintenance	13.4	0.020	—	—	—
**GO molecular function**			**GO molecular function**		
Structural constituent of bone	47.2	0.001	Inward rectifier potassium channel activity	24.6	0.007
cAMP response element binding protein binding	20.2	0.009	Voltage-gated ion channel activity	9.2	0.002
Enhancer binding	12.9	0.022	Cytokine activity	4.6	0.045
Anion transmembrane transporter activity	7.4	0.040	—	—	—
Transcription factor activity, RNA polymerase II	4.4	0.012	—	—	—
**GO cellular component**			**GO cellular component**		
Signal recognition particle	24.1	0.040	Voltage-gated potassium channel complex	9.6	0.038
Transcription elongation factor complex	8.4	0.011	axoneme	9	0.043
Trans-Golgi network membrane	6.9	0.049	Receptor complex	7.5	0.016
polysome	5.6	0.012	—	—	—
Transcription factor complex	3.1	<0.001	—	—	—
**Pathway analysis**			**Pathway analysis**		
Pantothenate and CoA biosynthesis	8.4	0.049	Retrograde endocannabinoid signaling	11.5	0.004
Arginine and proline metabolism	4.1	0.043	Cholinergic synapse	10.5	0.006
Glucagon signaling pathway	3.0	0.048	—	—	—
Glutamatergic synapse	2.6	0.038	—	—	—
PI3K-Akt signaling pathway	2.6	0.001	—	—	—

The most enriched terms of the dysregulated target mRNAs were indicative of that mRNA regulation has directed pattern which are related with the AD pathogenesis. In the 7 M brain, the most enriched GO and pathway terms for the up-regulated mRNAs are related with the immune activation (‘Macrophage colony-stimulating factor signaling pathway’ in BP, ‘Fc-gamma receptor I complex binding’ in MF, and ‘CSF1-CSF1R complex’ in CC), activation of inflammatory cascade (‘Complement and coagulation cascades’, ‘Staphylococcus aureus infection’, and ‘Hematopoietic cell lineage’ in Pathway analysis), cellular adhesion (‘Integrin complex’ in CC), and production of reactive oxygen species (‘Superoxide-generating NADPH oxidase activity’ in MF and ‘NADPH oxidase comple’x in CC) which are known as the major responses to overproduced Aβ in brain^2,3,9–11,30^. Also in the 12 M brain, most of the enriched terms for the up-regulated mRNAs are related with the immune acitivation (‘T cell activation via TCR contact with antigen bound to MHC molecule on APC’ and ‘Negative regulation of natural killer cell activation’ in BP, ‘C-X3-C chemokine binding’ in MF, and ‘Iimmunological synapse’ and ‘Phagocytic vesicle membrane’ in CC), inflammatory response (‘Positive regulation of tumor necrosis factor secretion’ and ‘Positive regulation of interleukin-1 production’ in BP and ‘Graft-versus-host disease’ and ‘Allograft rejection’ in pathway analysis), and cellular adhesion (‘Lipoteichoic acid binding’, ‘Fibronectin binding’, and ‘Integrin binding’ in MF, and ‘Integrin complex’ in CC).

In contrast, the most enriched GO and pathway terms for the down-regulated mRNAs in the 7 M brain are commonly related with progenitor self-renewal and neuronal differentiation (‘Muscle cell fate commitment’, ‘Negative regulation of muscle hyperplasia’, and ‘Schwann cell proliferation’ in BP) and maintenance (‘Regulation of store-operated calcium entry’ and ‘Mitochondrial genome maintenance’ in BP, ‘Structural constituent of bone’ and ‘cAMP response element binding protein binding’ in MF, ‘Trans-Golgi network membrane’ and ‘Transcription elongation factor complex’ in CC, and ‘Pantothenate and CoA biosynthesis’, ‘Arginine and proline metabolism’, and ‘Glucagon signaling pathway’ in pathway analysis). Additionally, the down-regulated mRNAs in 12 M brain were enriched in synapse function and preservation of neuronal networks (‘Positive regulation of action potential’ in BP, ‘Inward rectifier potassium channel activity’ and ‘Voltage-gated ion channel activity’ in MF, ‘Voltage-gated potassium channel complex’ and ‘Receptor complex’ in CC, and ‘Retrograde endocannabinoid signaling’ and ‘Cholinergic synapse’ in pathway analysis), which is a competent finding with the overt manifestation of AD phenotypes at twelve months^31^.

## Discussion

This study demonstrated the altered expression of circRNAs and their possible epigenetic regulatory effect at different time points in the brain of an AD model. Although a very small number of circRNAs had a significantly altered expression (0.7% in 7 M and 0.1% in 12 M), they targeted large number of mRNAs by circRNA–miRNA–mRNA interaction. Dysregulated circRNAs were associated with a significantly higher frequency of the relevant dysregulation of the downstream target mRNAs, after adjusting the effect of dysregulated upstream miRNAs. Additionally, gene ontology and pathway analyses demonstrated that the mRNAs of the differently expressed circRNA–miRNA–mRNA interactions might be closely involved in the pathomechanism of AD. Numerous studies have evaluated the miRNAs’ epigenetic regulatory effect on disease relevant genes^1,2,8–15^. However, this study demonstrated that circRNAs might have more significant regulatory effect on the disease relevant genes than miRNAs.

In this study, we found no significant change in the frequency of dysregulation in the downstream target mRNAs of the differentially expressed miRNAs. This negative association might be due to the high complexity of miRNA-mRNA interactions and the high temporal variability of miRNA expression levels in the brain^16–19^. Additionally, no significant change was observed in the frequency of dysregulation in the downstream target miRNAs of the differentially expressed circRNAs. This might be attributable to that the regulatory effect of circRNAs on miRNAs might not necessarily result in an alteration of target miRNA expression level^25^. Some properties of circRNAs might explain the regulatory effect of circRNA on target mRNAs that exceeds the effect of miRNA. First, due to substantial complexity in the circRNA−miRNA−mRNA interactions, a single dysregulation of a miRNA can hardly induce a significant dysregulation in downstream target genes, as they are simultaneously regulated by hundreds of other upstream RNAs^18,19^. Second, as circRNAs are more stable than miRNAs in CNS^21,29^, their altered expression can sufficiently buffer the fickle changes of miRNAs’ effect and modulate the overall gene expression profile in response to a certain disease process^20–22^.

No circRNAs except for the mmu_circRNA_29980 was consistently dysregulated in 7 M and in 12 M brains, suggesting a high time specificity in the regulation of the circRNA expression, which is consistent with the findings of the previous studies. This might indicate that circRNA expression is dynamically regulated according to each stage of the disease progression to exert an epigenetic regulatory effect on their downstream targets relevantly to the disease pathomechanism^28,29^.

Gene ontology and pathway analyses demonstrated that the mRNAs of the differently expressed circRNA–miRNA–mRNA regulatory networks might be closely involved in the pathomechanism of AD. The enriched terms of up-regulated target mRNAs in both 7 M and 12 M brains were commonly associated with the immune activation, activation of inflammatory cascade, and cellular adhesion. Considering that Tg2576 mice show progressive accumulation of amyloid-b42 (Aβ42) in brain which becomes evident at seven months, these up-regulated mRNAs might be involved in the major responses to overproduced Aβ in brain^2,3,9–11,30^. In contrast, the enriched terms of down-regulated target mRNAs in both time points were associated with the progenitor self-renewal, neuronal differentiation and maintenance. These findings represent the ongoing deterioration of metabolic homeostasis and subsequent neuronal degeneration during the disease progression^2,3,8,10,32^. Additionally, the enriched terms of down-regulated target mRNAs 12 M were associated with synapse function and preservation of neuronal networks, which is competent with the overt manifestation of AD phenotypes at twelve months^3,4,8,31^. Therefore, the results of the gene ontology and pathway analyses suggests that the altered expression of circRNAs might direct the overall gene expression profile relevantly to a certain disease process in cellular, network, and phenotypical (cognitive) levels via circRNA-miRNA-mRNA networks. In this regard, the dysregulated circRNAs might involve in the AD pathogenesis in disease stage specific manners.

There are several limitations in this study that should be acknowledged. First, because of the large number of genes analyzed, validation of their expression ratios with quantitative PCR analyses was performed for the limited number of the differentially expressed circRNAs and mRNAs. Second, we only evaluated the association between the dysregulated circRNA, miRNA, and mRNAs and did not directly validate the regulatory interactions among them. Especially, quantitative analyses of the association between the changes of the MRE expression in dysregulated circRNAs and the expression levels of their target mRNAs were not performed, due to the high complexity of circRNA-miRNA-mRNA interactions. Third, the role of the circRNA−miRNA−mRNA regulatory network in the AD pathomechanism was not validated but only predicted by bioinformatics methods. Future studies should endeavor to confirm the regulatory interaction among the circRNA, miRNA, and mRNA and network verify a specific circRNA−miRNA−mRNA regulatory network with a sufficient pathomechanistic role in AD, which might serve as a disease marker and potential therapeutic target.

## Materials and Methods

### Study design

Tg2576 AD transgenic mice (Taconic, Hudson, NY) were used in this study, which highly express the human 695-amino acid isoform of amyloid precursor protein containing the Swedish double mutation causing early-onset AD. Tg2576 mice show increased amyloid-b42 (Aβ42) expression in brain at seven months and manifests AD phenotypes at twelve months^1,31^. Hence, four male Tg2576 mice with seven months of age (7 M) and four male with twelve months of age (12 M) were chosen and compared with the normal male C57BL/6 mice. All animals were managed with standardized procedures approved by the Institutional Animal Care and Use Committee of Seoul National University Hospital.

### Tissue preparation and microarray

Animals were sacrificed and their brain samples were taken. Control mice of the same number were sacrificed at the same age as the Tg2576 mice. The brains were obtained from each mouse and immediately stored at −80 °C. For microarray analysis, total RNAs were extracted using TRIZOL reagent (Invitrogen, NY, USA) and purified by an RNeasy Mini Kit (Qiagen, Hilden, Germany). RNA quantity was measured with a Nanodrop ND-1000 (Thermo Fisher Scientific, MA, USA) and quality checked by an Agilent 2100 Bioanalyzer (Agilent Technologies, CA, USA)^1^.

For circRNA microarray, the RNAs were treated with RNase R (Epicenter, WI, USA) to remove linear RNAs and enrich circRNA. The enriched circRNA samples were amplified and transcribed into fluorescent cRNA using a random priming method using Arraystar Super RNA Labeling Kit (Arraystar, Rockville, MD, USA). The labeled cRNAs were hybridized onto the Arraystar Mouse circRNA Array V2 (8 × 15 K, Arraystar). After the slides were washed, the arrays were scanned by an Agilent Scanner G2505C (Agilent Technologies). The miRNA and mRNA microarray data were also obtained using the Agilent Mouse miRNA Microarray 8 × 15 K kit and Agilent Mouse Gene Expression Microarray 4 × 44 K kit respectively, according to the manufacturer’s protocol (Agilent Technologies)^1^.

Agilent Feature Extraction software (Agilent Technologies) was used to analyze the acquired array images. Quantile normalization and subsequent data processing were performed with the Agilent’s GeneSpring Software (Agilent Technologies). Mann–Whitney test was used to detect differentially expressed circRNAs and mRNAs between the Tg2576 mice and the controls by fold-changes of ≥1.5 and *P*-values of < 0.05 with relatively small false discovery rates (FDRs). Scatter plots (for circRNAs and mRNAs), volcano plot, and hierarchical clustering (for circRNAs) were used to demonstrate the expression pattern of circRNAs and mRNAs.

### Analysis of circRNA–miRNA–mRNA regulatory interaction

The regulatory effect of miRNAs on their target mRNAs was separately evaluated for the up- and down-regulated miRNAs in each time point (7 M and 12 M). For each dysregulated miRNA, potential target mRNAs were predicted by the combination of TargetScan and miRanda^14,22^. Expression profiles of those potential target mRNAs were obtained from the microarray data and the change in the frequency of relevant dysregulation (down-regulation of target mRNAs for up-regulated miRNAs or up-regulation of target mRNAs for down-regulated miRNAs) in those target mRNAs were evaluated.

To evaluate whether the dysregulate circRNAs alter the expression level of their target miRNAs, up to five target miRNAs for each differentially expressed circRNA were identified by microRNA response element (MRE) analysis for the circRNA sequence. MRE sequence analysis was performed using miRNA target prediction software (Arraystar) based on TargetScan (http://www.targetscan.org) and miRanda (http://www.microrna.org) algorithms^18,26^. Using the microarray data, the change in the frequency of relevant dysregulation in those target miRNAs were evaluated.

To demonstrate whether the dysregulated circRNAs alter the expression level of their downstream target mRNAs by circRNA–miRNA–mRNA interactions. We found that the dysregulated circRNAs does not alter the frequency of relevant dysregulation in their target miRNAs^25–27^. However, as circRNAs inhibit or buffer the miRNA’s effect of suppressing its target mRNAs, we speculated that an up-regulated circRNA might exert a disinhibitory effect on its downstream targets mRNAs and a down-regulation of a circRNA might induce a hyper-inhibition on its downstream target mRNAs^17,18^. Therefore, we evaluated whether dysregulated circRNAs can induce a significant change in the frequency of relevant dysregulation in the downstream target mRNAs^25,26^.

### PCR analysis

Based on the microarray data, three circRNAs and three mRNAs were randomly selected from each of the top ten (five up-regulated and five down-regulated) most highly dysregulated circRNAs and mRNAs in 7 M and 12 M. For PCR analysis, two brain samples were pooled into one RNA sample as a unit. cDNAs were synthesized from 0.5 μg of total RNA of brain tissues by reverse transcription. Standard curves were prepared using 2 × SuperArray PCR master mix (Arraystar) according to the manufacturer’s protocol. The relative expression ratio of each circRNA and mRNA was calculated with the Rotor-Gene Real-Time Analysis Software 6.0 (Qiagen), using the housekeeping gene, *Gapdh*, expression for normalization. All real-time reactions were performed in triplicate^27^.

### Specific circRNA–miRNA–mRNA regulatory network

We found that the regulatory effect of circRNAs on their target miRNAs does not changes the frequency of the target miRNA dysregulation but is associated with a higher frequency of relevant dysregulation in the downstream target mRNAs^25^. Therefore, we constructed differentially expressed circRNA–miRNA–mRNA regulatory networks comprised of a dysregulated circRNA, its target miRNA, and miRNA’s downstream target mRNA which is also dysregulated in the same direction with the upstream circRNA dysregulation, without considering whether the miRNA expression level was altered^25–27^. Additionally, visualizations of the differentially expressed circRNA–miRNA–mRNA regulatory networks were performed using Cytoscape 3.4.0^33^.

### Gene ontology and pathway analysis

To demonstrate the pathophysiologic role of the differentially expressed circRNA–miRNA–mRNA regulatory network in AD pathophysiology, gene ontology and pathway analyses were performed for the target mRNAs. The gene ontology domains included Biological process (BP), Molecular function (MF), and Cellular component (CC) and were obtained using Database for Annotation, Visualization and Integrated Discovery (DAVID; http://www.david.abcc.ncifcrf.gov/)^34,35^. Pathway analysis was performed using the Kyoto Encyclopedia of Genes and Genomes (KEGG, http://www.genome.jp/kegg) database^36^. In both analyses, Fisher’s exact or chi-squared test with FDR were used, where a GO term or KEGG pathway with *P*-value < 0.05 and FDR < 0.05 was considered statistically significant. The top five enriched GO terms and pathways of the differentially expressed target mRNAs were ranked by fold enrichment score.

### Statistical analysis

Data were reported as number (percentage) or mean ± standard deviation. Mann–Whitney *U* test was used to detect differentially expressed circRNAs, miRNAs, and mRNAs between the two groups by fold-changes of ≥1.5 and *P*-values ≤ 0.05. Changes in the frequency of relevant dysregulation in the downstream target RNAs were evaluated using Pearson’s chi-square test. To evaluate factors associated with significant dysregulation of mRNA, logistic regression analyses including parameters with *P*-values < 1.20 in univariate analyses were performed. SPSS (version 23.0; SPSS Inc., Chicago, IL, USA) was used to all statistical analyses. *P*-values < 0.05 were considered statistically significant.

## Supplementary information

Dataset 1

Dataset 2

Dataset 3

Dataset 4

Dataset 5

Dataset 6

Dataset 7

## Data Availability

Full microarray, gene ontology, and pathway analysis analysis data of this study is available in the Supplemental Datasets.
